# Phantom Limb Pain: Mechanisms and Treatment Approaches

**DOI:** 10.1155/2011/864605

**Published:** 2011-08-14

**Authors:** Bishnu Subedi, George T. Grossberg

**Affiliations:** Department of Neurology & Psychiatry, Saint Louis University School of Medicine, St. Louis, MO 63104, USA

## Abstract

The vast amount of research over the past decades has significantly added to our knowledge of phantom limb pain. Multiple factors including site of amputation or presence of preamputation pain have been found to have a positive correlation with the development of phantom limb pain. The paradigms of proposed mechanisms have shifted over the past years from the psychogenic theory to peripheral and central neural changes involving cortical reorganization. More recently, the role of mirror neurons in the brain has been proposed in the generation of phantom pain. A wide variety of treatment approaches have been employed, but mechanism-based specific treatment guidelines are yet to evolve. Phantom limb pain is considered a neuropathic pain, and most treatment recommendations are based on recommendations for neuropathic pain syndromes. Mirror therapy, a relatively recently proposed therapy for phantom limb pain, has mixed results in randomized controlled trials. Most successful treatment outcomes include multidisciplinary measures. This paper attempts to review and summarize recent research relative to the proposed mechanisms of and treatments for phantom limb pain.

## 1. Introduction

The concept of phantom limb pain (PLP) as being the pain perceived by the region of the body no longer present was first described by Ambrose Pare, a sixteenth century French military surgeon [1]. Silas Weir Mitchell, a famous Civil War surgeon in the nineteenth century, coined the term “phantom limb pain” and provided a comprehensive description of this condition [2]. It continues to remain a poorly understood and difficult to treat medical condition. A recent study estimated that there were about 1.6 million people with limb loss in the USA in 2005 and this number was projected to increase by more than double to 3.6 million by the year 2050 [3]. Vascular problems, trauma, cancer, and congenital limb deficiency are among the common causes of limb loss. The number of traumatic amputations has also increased since the beginning of conflict in Iraq and Afghanistan [4]. The incidence of PLP has been reported to range from 42.2 to 78.8% in patients requiring amputation [5–8].

Stump pain is described as the pain in the residual portion of the amputated limb whereas phantom sensations are the nonpainful sensations experienced in the body part that no longer exists [6, 7]. Superadded phantom sensations are touch and pressure-like sensations felt on the phantom limb from objects such as clothing [9]. Risk factor for PLP are shown in Table 1. Recent studies report the prevalence of PLP to be more common among upper limb amputees than lower limb amputees. It was also reported to be more common among females than males [10, 11]. A survey reported greater overall average pain intensity and interference in females than males and females endorsed significantly greater catastrophizing, use of certain pain-coping strategies, and beliefs related to several aspects of pain resulting in poor adjustment [12]. Larger population studies are needed for more definite establishment of the risks associated due to the site of involved limb or gender of the patient in development of PLP. Phantom sensations and pain have been reported following amputation of different body parts including the eyes, teeth, tongue, nose, breast, penis, bowel, and bladder but the most common occurrence is following limb amputation [4]. The phantom pain and sensation may have its onset immediately or years after the amputation. There are reports of two peak periods of onset, the first within a month and the second a year after amputation [7]. The prevalence is reported to decrease over time after amputation [10, 11]. PLP has been reported in people with congenital absence of limbs [13]. Tingling, throbbing, piercing, and pins and needles sensations were among the most commonly described types of pain [13]. The rate of phantom pain or sensation was not reported to be higher in people with bilateral limb amputation than those with single limb amputation [14]. A significant association has been reported between the PLP and residual limb pain [15]. The presence of preamputation pain is also reported to increase the risks of developing PLP [16]. It is likely that stress, anxiety, depression, and other emotional triggers contribute to the persistence or exacerbation of PLP. A study has found that amputees with depressive symptoms were more likely to characterize their pain as more severe than those without depressive symptoms [17].

## 2. Mechanisms

PLP was once thought to be primarily a psychiatric illness. With the accumulation of evidence from research over the past decades, the paradigm has shifted more towards changes at several levels of the neural axis, especially the cortex [18]. Peripheral mechanisms and central neural mechanisms are among the hypotheses that have gained consensus as proposed mechanisms over the recent years. Proposed mechanisms to explain phantom limb pain are shown in Table 2. However none of these theoretical constructs appears to be able to explain the phenomenon of PLP independently and many experts believe that multiple mechanism are likely responsible.

### 2.1. Peripheral Mechanism

During amputation, peripheral nerves are severed. This results in massive tissue and neuronal injury causing disruption of the normal pattern of afferent nerve input to the spinal cord. This is followed by a process called deafferentation and the proximal portion of the severed nerve sprouts to form neuromas [18]. There is an increased accumulation of molecules enhancing the expression of sodium channels in these neuromas that results in hype-excitability and spontaneous discharges [19]. This abnormal peripheral activity is thought to be a potential source of the stump pain, including phantom pain [18]. Studies reporting the reduction of phantom pain with drugs blocking the sodium channels lend further support to this theory [20, 21]. However, this cannot explain the mechanism of PLP in patients with congenital absence of limbs [4, 18].

### 2.2. Central Neural Mechanisms

#### 2.2.1. Changes at the Level of Spinal Cord

The axonal sprouts at the proximal section of the amputated peripheral nerve form connections with the neurons in the receptive field of the spinal cord. Some neurons in the areas of spinal cord that are not responsible for pain transmission also sprout into the Lamina II of the dorsal horn of the spinal cord which is the area involved in the transmission of nociceptive afferent inputs [18, 19]. This is followed by increased neuronal activity, expansion of the neuronal receptive field, and hyperexcitability of other regions. This process is called central sensitization. During this process, there is also an increase in the activity at NMDA receptors mediated by neurotransmitters such as substance P, tachykinins, and neurokinins at the dorsal horn of the spinal cord [22]. This is followed by a phenomenon called the “windup phenomenon” in which there is an upregulation of those receptors in the area [22]. This process brings about a change in the firing pattern of the central nociceptive neurons. The target neurons at the spinal level for the descending inhibitory transmission from the supraspinal centers may be lost. There also may be a reduction in the local intersegmental inhibitory mechanisms at the level of the spinal cord, resulting in spinal disinhibition and nociceptive inputs reaching the supra spinal centers. This lack of afferent input and changes at the level of the spinal cord have been proposed to result in the generation of PLP [22–24].

#### 2.2.2. Changes at the Level of the Brain

Cortical reorganization is perhaps the most cited reason for the cause of PLP in recent years. During reorganization, the cortical areas representing the amputated extremity are taken over by the neighboring representational zones in both the primary somatosensory and the motor cortex [18, 25, 26]. The process and extent of cortical reorganization have been studied in both animal and human models following amputation and deafferentation. Cortical reorganization partly explains why the afferent nociceptive stimulation of neurons within the stump or surrounding area produces the sensation in the missing limb [4, 27]. The extent of cortical reorganization has been found to be directly related to the degree of pain and the size of the deafferentiated region. Multiple imaging studies have correlated greater extent of somatosensory cortex involvement with more intense phantom limb experience [4, 28–30].

Another proposed mechanism of PLP is based on the “body schema” concept that was originally proposed by Head and Holmes in 1912. The body schema can be thought of as a template of the entire body in the brain and any change to the body, such as an amputation, results in the perception of a phantom limb [31]. A further expansion of the body schema concept is the “neuromatrix and neurosignature” hypothesis proposed by Ronald Melzack in 1989. The neuromatrix can be conceptualized as a network of neurons within the brain that integrates numerous inputs from various areas including somatosensory, limbic, visual, and thalamocortical components. It then results in an output pattern that evokes pain or other meaningful experiences. The term “neurosignature” was proposed by Melzack to refer to the patterns of activity generated within the brain that are continuously being updated based upon one's conscious awareness and perception of the body and self. The deprivation of various inputs from the limbs to the neuromatrix causes an abnormal neurosignature to be produced that results in the generation of PLP [32–34].The other hypothesis relative to the mechanism of PLP has been derived from the research into illusory perceptions. It has been shown that the parietal and frontal lobes are also involved besides the primary somatosensory cortex in the perception of the abnormal somatosensory phenomenon [35]. Painful sensations, such as PLP, may be related to the incongruence of motor intention and sensory feedback and a corresponding activation of the parietal and frontal brain areas [36, 37].

### 2.3. Psychogenic Mechanism

The assumption that PLP is of psychogenic origin has not been supported in the recent literature even though stress, anxiety, exhaustion, and depression are believed to exacerbate PLP [38]. A cross-sectional study found that amputation in people with personality traits characterized by passive coping styles and catastrophizing behavior was found to be associated with the development of PLP independent of anxiety and depression [39]. Most research on the relationship between psychological symptoms and PLP has been retrospective and cross sectional rather than longitudinal and thus limited inferences can be derived from these studies.

## 3. Treatment

A number of different therapies relying on different principles have been proposed for the management of PLP as shown in Table 3. However, specific treatment guidelines are yet to evolve and most successful measures employ multidisciplinary approaches in the management of pain and in rehabilitation [40].

### 3.1. Pharmacological Approaches

#### 3.1.1. PreEmptive Analgesia and Anesthesia

Preemptive use of analgesics and anesthetics during the preoperative period is believed to prevent the noxious stimulus from the amputated site from triggering hyperplastic changes and central neural sensitization which may prevent the amplification of future impulses from the amputation site [42]. However, the results of the studies in this area have not been definitive. A recent study reported the decrease in PLP at six months following amputation when optimized epidural analgesia or intravenous patient controlled analgesia was started between 48 hours preoperatively and 48 hours postoperatively [20]. Prolonged postoperative perineural infusion of ropivacaine 0.5% was reported to prevent or reduce PLP and sensations after lower extremity amputation [21]. Ketamine, however, was not found to significantly reduce acute central sensitization or the incidence and severity of postamputation pain [43]. A randomized controlled double-blind trial comparing epidural infusions between a group receiving ketamine and bupivacaine and another receiving ketamine and saline following intrathecal or epidural anesthetic for surgery showed no significant difference between the two groups but much less pain at one year was reported in both groups compared to other comparable studies [44].

#### 3.1.2. Acetaminophen and Nonsteroidal Anti-Inflammatory Drugs (NSAIDs)

A cross sectional study found that acetaminophen and NSAIDs were the most common medications used in the treatment of PLP [45]. The analgesic mechanism of acetaminophen is not clear but serotonergic and multiple other central nervous system pathways are likely to be involved [46]. NSAIDs inhibit the enzymes needed for the synthesis of prostaglandin and decrease the nociception peripherally and centrally [47].

#### 3.1.3. Opioids

Opioids bind to the peripheral and central opioid receptors and provide analgesia without the loss of touch, proprioception, or consciousness. They may also diminish cortical reorganization and disrupt one of the proposed mechanisms of PLP [4]. Randomized controlled trials have demonstrated the effectiveness of opioids (oxycodone, methadone, morphine, and levorphanol) for the treatment of neuropathic pain including PLP. Comparative trials have also shown benefit with opioids when compared with tricyclic antidepressants and gabapentin though the opioids were associated with more frequent side effects [48]. The total amount of opioid required to achieve analgesia may be less when used together with other agents, such as tricyclic antidepressants or anticonvulsants, which also have use in neuropathic pain modulation. Tramadol, a weak opioid and a mixed serotonin-noradrenalin reuptake inhibitor, has also been used in the treatment of PLP [4, 49].

#### 3.1.4. Antidepressants

Tricyclic antidepressants are among the most commonly used medications for various neuropathic pains including PLP. The analgesic action of tricyclic antidepressant is attributed mainly to the inhibition of serotonin-norepinephrine uptake blockade, NMDA receptor antagonism, and sodium channel blockade [50]. The role of tricyclic antidepressants is well established in other neuropathic pain conditions, but the results are mixed relative to their role on PLP [51]. A recent study reported excellent and stable PLP control with an average dose of 55 mg of amitryptline, but there are others in which tricyclic antidepressants failed to effectively control the pain. [49, 52]. Nortriptyline and desipramine have been found to be equally effective and with less side effects compared to amitriptyline [53]. A small case series demonstrated the effectiveness of mirtazapine, an alpha 2 receptor antagonist with fewer side effects than tricyclic antidepressants in the treatment of PLP [54]. There are case reports relative to the efficacy of duloxetine, a NE and serotonin receptor inhibitor, in the treatment of PLP [55]. Even though there may be a role for the use of SSRI and SNRI in the treatment of neuropathic pain, the evidence is very limited and further research is needed [56].

#### 3.1.5. Anticonvulsants

Gabapentin has shown mixed results in the control of PLP with some studies showing positive results while others not showing efficacy [57–59]. Carbamazepine has been reported to reduce the brief stabbing and lancinating pain associated with PLP. Oxcarbazepine and pregabalin may also play a role in the treatment of PLP, but further studies are required [4, 60].

#### 3.1.6. Calcitonin

The mechanism of action of calcitonin in treatment of PLP is not clear. Studies relative to its therapeutic role have been mixed [61, 62].

#### 3.1.7. NMDA Receptor Antagonist

The mechanism of action of NMDA receptor antagonism in PLP is not clear. Memantine has shown some benefits in some case studies but controlled trials have shown mixed results [63, 64]. A review concluded that memantine may be useful soon after amputation rather than for use in chronic neuropathic pain conditions [65].

#### 3.1.8. Other Medications

The beta blocker propranolol and the calcium channel blocker nifedipine have been used for the treatment of PLP [60]. However, their effectiveness is unclear and further studies are needed. Flupirtine, an NMDA antagonist and potassium channel agonist, has been reported to be effective when used together with opioids in cancer-related neuropathic pain but needs further studies for other etiologies [66].

### 3.2. Nonpharmacological Treatment

#### 3.2.1. Transcutaneous Electrical Nerve Stimulation (TENS)

Transcutaneous electrical nerve stimulation has been found to be helpful in PLP [40]. Historically, there have been multiple studies showing the effectiveness of TENS of the contralateral limb versus ipsilateral to decrease PLP [67]. Though there is no strong evidence, low-frequency and high-intensity TENS is thought to be more effective than other doses [68]. TENS devices are portable, are easy to use, and have few side effects or contraindications.

#### 3.2.2. Mirror Therapy

Mirror therapy was first reported by Ramachandran and Rogers-Ramachandran in 1996 and is suggested to help PLP by resolving the visual-proprioceptive dissociation in the brain [69, 70]. The patient watches the reflection of their intact limb moving in a mirror placed parasagittally between their arms or legs while simultaneously moving the phantom hand or foot in a manner similar to what they are observing so that the virtual limb replaces the phantom limb. Simian studies have shown the existence of mirror neurons in the brain which fire both at times when an animal performs an action or observes an action [71]. Similar homologous neurons have also been discovered in humans [72]. The presence of mirror neurons in the brain is also supported by the phenomenon of tactile sensation in the phantom limb elicited by touching the virtual image of the limb in the mirror [73]. When a person with an intact limb observes a person with amputation, he can only “empathize about the amputation” rather than “feel it himself” because of the null input to the mirror neurons from his intact limb. However, a person with an amputation does not receive such null input as the limb is amputated and this results in the activation of mirror neurons which create a perception of tactile sensation. Consequently, since the activation of these mirror neurons modulates somatosensory inputs, their activation may block protopathic pain perception in the phantom limb [72, 73]. A randomized controlled trial of mirror therapy in patients with lower leg amputation has shown significant benefit of PLP versus the control group [74]. Another controlled trial, however, reported that the mirror condition only elicited a significantly greater number of phantom limb movements than the control condition but did not attenuate phantom limb pain and sensations any more than the control condition [75].

#### 3.2.3. Biofeedback, Integrative, and Behavioral Methods

Although there are earlier reports suggesting temperature biofeedback to be helpful for burning sensation of PLP, there is no specific evidence to match specific types of PLP with specific biofeedback techniques [76]. There is also a case report of visual feedback helpful in reduction of phantom pain [74]. Guided imagery, relaxation techniques, and hypnosis have been employed in the treatment of different neuropathic pains and may also be useful for PLP [28, 77, 78]. There are case reports of the beneficial effect of acupuncture for PLP [79, 80]. The effectiveness of cognitive behavioral therapy in neuropathic pain syndromes has been reported in a number of case studies [81, 82].

#### 3.2.4. Surgical Intervention

Surgical interventions are usually employed when other treatment methods have failed. A case report relates the effectiveness of lesioning the dorsal root entry zone (DREZ) on upper limb phantom pain resulting from brachial plexus avulsions [83]. Another case report showed that, for selected patients, who have not obtained adequate relief with medical management, spinal cord stimulation was found to be effective [84]. Case reports of improvement of PLP with deep brain stimulation of the periventricular gray matter and thalamic nuclei have been published [85]. Motor cortex stimulation was also found to be helpful in a case of PLP [86].

#### 3.2.5. Electroconvulsive Therapy

A case report of positive outcome has been published even though the mechanism and role of ECT relative to PLP is not well understood [87].

## 4. Conclusion

PLP is a relatively common and disabling entity. We have learned much about the pathophysiology and management of PLP since it was first described about five centuries ago. However, there is still no one unifying theory relative to the mechanism of PLP. Specific mechanism-based treatments are still evolving, and most treatments are based on recommendations for neuropathic pain. The evolution of the mechanistic hypothesis from body schema and neuropathic theories to the recently proposed role of mirror neurons in the mechanism of pain have added to our understanding of PLP. Further research is needed to elucidate the relationship between the different proposed mechanisms underlying PLP. A synthesized hypothesis explaining the phenomenon of PLP is necessary in the future for the evolution of more specific mechanism-based treatment recommendations.

## Figures and Tables

**Table 1:** Risk factors for phantom limb pain.

Female sex
Upper extremity amputation
Presence of preamputation pain
Residual pain in remaining limb
Time after amputation

**Table 2:** Proposed theoretical mechanisms to explain phantom limb pain.

(1) Pheripheral mechanism	
Stump and neuroma hyperactivity	

(2) Central neural mechanisms	
Spinal cord sensitization and changes	
Cortical reorganization and cortical-motor sensory dissociation	
Body schema, neuromatrix and neurosignature hypothesis	

(3) Psychogenic mechanism	

**Table 3:** Treatments for phantom limb pain.

Pharmacotherapy	Surgical/invasive procedures	Adjuvant therapy
Opioids	Stump revision	Transcutaneous nerve stimulation
Morphine	Nerve block	Mirror therapy
Tramadol	Neurectomy	Biofeedback
Tricyclic Antidepressants	Rhizotomy	Temperature biofeedback
Amitriptyline	Cordotomy	Electro myographic biofeedback
Nortriptyline	Lobectomy	Massage
Imipramine	Sympathectomy	Ultrasound
Desipramine	CNS stimulation	Physiotherapy
AntiConvulsants	Spinal cord stimulation	Sensory discrimination training
Carbamazepine	Deep brain/thalamus stimulation	Prosthesis training
Oxcarbazepine	Cortical stimulation	Cognitive behavioral pain management
Gabapentin		Electroconvulsive therapy
Pregabalin		
Sodium channel blockers		
Lidocaine		
Bupivacaine		
Mexiletine		
NMDA receptor antagonist		
Memantine		
Ketamine		

Adapted from [4, 41].
